# Use of a Nonimmersive Virtual Reality System for Clinical Thinking in Obstetric Nursing Education: Mixed Methods Study

**DOI:** 10.2196/80951

**Published:** 2025-11-24

**Authors:** Sixing Liu, Jiaxun Yang, Caihong Zhang, Jing Zhang, Qin Wang, Rong Zhou, Ziran Zhao, Shan Ren, Huanying Yi, Honghua Guo, Jieqiong Xia

**Affiliations:** 1 School of Nursing Hainan Medical University Haikou, Hainan China; 2 School of Nursing Hangzhou Normal University Hangzhou China; 3 School of Biomedical Information and Engineering Hainan Medical University Haikou China; 4 Department of Otolaryngology Head and Neck Surgery The First Affiliated Hospital of Zhengzhou University Zhengzhou China

**Keywords:** Nonimmersive Virtual Reality System for Clinical Thinking in Obstetric Nursing, NIVRSCTON, obstetric nursing training, clinical thinking, salutogenesis, virtual reality, obstetrics, nursing

## Abstract

**Background:**

Traditional obstetric nursing training faces limitations in inadequate interactivity and nonrepeatable demonstrations, limiting students’ development of clinical thinking. Virtual reality (VR) offers a solution for complex health care education, enhancing nursing students’ clinical thinking.

**Objective:**

This study applied the Nonimmersive Virtual Reality System for Clinical Thinking in Obstetric Nursing (NIVRSCTON), grounded in salutogenesis theory, to examine its effects on nursing students.

**Methods:**

The NIVRSCTON was applied under the auspices of the Nursing Virtual Teaching Hub in the Coastal Area (NVTHCA). In September 2023, a convenience sample of 88 undergraduate nursing students from 4 partner institutions participated in the study. A single-group pre-post design and an explanatory sequential mixed methods design were used to measure changes in clinical thinking ability following the training and to assess the system’s performance. The quantitative assessment tools included the general information questionnaire, the Clinical Thinking Ability Evaluation Scale (CTAES), and the Evaluation Instrument for Virtual Reality System (EIVRS). Each student was required to submit one reflective journal developed in accordance with the Bass model. Quantitative data were analyzed using IBM SPSS (version 22.0), and qualitative data were thematically coded using NVivo (version 12; QSR International Pty Ltd).

**Results:**

After the NIVRSCTON training was completed, the students’ overall clinical thinking score increased from 49.08 (SD 11.30) to 80.50 (SD 11.01), indicating a significant improvement (*t*_87_=–18.76; Cohen *d*=–2.82, 95% CI –34.74 to –28.08). All clinical thinking dimension scores improved, and the improvements were all statistically significant (*P*<.001). Critical thinking scores increased from 13.98 (SD 3.76) to 24.77 (SD 3.11; *t*_87_=–20.37; Cohen *d*=–3.13, 95% CI –11.85 to –9.74), system thinking scores increased from 26.82 (SD 6.40) to 44.51 (SD 6.24; *t*_87_=–19.18; Cohen *d*=–2.80, 95% CI –19.53 to –15.86), and evidence-based thinking scores improved from 18.10 (SD 4.40) to 27.31 (SD 4.61; *t*_87_=–13.42; Cohen *d*=–2.04, 95% CI –10.57 to –7.84). The variable *df* is all 87. In terms of application effectiveness, the students provided the following ratings: 0.82 (SD 0.15; rated as good) for interface design, 0.82 (SD 0.15; rated as good) for technical performance, 0.83 (SD 0.14; rated as good) for learning content, and 0.85 (SD 0.15; rated as excellent) for learning function. The overall evaluation was 0.82 (SD 0.15; rated as good). Qualitative data revealed that the training not only improved the clinical thinking and decision-making skills of the nursing students but also fostered their professional attitudes, values, and emotions.

**Conclusions:**

NIVRSCTON training enhances students’ clinical thinking and professionalism. It was well received, confirming its effectiveness. As an obstetric nursing teaching tool, it enhances clinical thinking and professional competence. It may also promote equity and access in nursing education, offering an innovative model for digital nursing education.

## Introduction

In response to the aging of the global population and to avoid the harms of low fertility, the Chinese government introduced the universal 2-child policy in 2016, followed by the 3-child policy [1,2]. Consequently, the proportion of advanced maternal age women and those with gestational complications or concomitant medical disorders has risen sharply [3]. As a result, the demands on obstetric nursing services and the need for a highly qualified obstetric nursing workforce have increased abruptly [4,5].

Unlike New Zealand, the United Kingdom, and Finland (where midwifery is an independent profession [6,7]), midwifery in China remains a subspecialty of nursing. In the past, midwifery education in China was conducted mainly at the technical secondary and college levels; it was not until 2017, following a surge in demand for obstetric nurses after the implementation of the 2-child policy, that the government established undergraduate midwifery programs [8].

However, the boundary between midwifery and nursing remains ambiguous. In most Chinese hospitals, obstetric care is provided by both nurses and midwives, and nursing graduates may work in obstetric units. Moreover, as expectations for obstetric care among women and their families increase and as the number of advanced maternal age women increases, the specificity and complexity of obstetric nursing practice pose significant challenges. This trend underscores the need for nursing students to develop strong clinical thinking skills in this area.

Clinical thinking includes systems thinking, critical thinking, and evidence-based thinking. It is a core competency for nursing students to navigate complex clinical situations and make effective decisions. Strong clinical thinking enables nurses to provide timely, patient-centered, and individualized care, while enhancing patient safety [9,10]. However, studies have shown that there is a significant gap in the development of clinical thinking among obstetric nursing students in China [11]. First, heightened awareness of privacy and self-protection among Chinese laboring women reduces students’ opportunities to practice clinical thinking in real clinical settings [12]. Second, traditional obstetric nursing training is often ineffective because of 3 main factors: poor visibility of procedures, limited opportunities for teacher-student interactions, and a lack of repeatable demonstrations [13]. Consequently, students gain little experience in emergency management, leaving them unprepared to rapidly synthesize patient data, identify complications, and choose effective interventions during abnormal labor or obstetric crises. This results in delayed clinical decisions and suboptimal nursing measures. Consequently, fostering clinical thinking in obstetric nursing practice has become imperative for advancing the quality of care provided to women.

In the digital era, virtual reality (VR) is being extensively adopted in obstetric nursing education [14]. Jeong and Lim [14] developed a VR module on normal vaginal delivery, which was administered to third-year nursing students after 1-2 weeks of clinical observation. Their results indicated that the module significantly improved students’ self-directed learning, teamwork, and clinical decision-making skills, ultimately enhancing their readiness for clinical rotation. High-fidelity, service-oriented VR training environments have the potential to effectively address the inherent limitations of traditional training—such as physical constraints and scarce equipment—while offering distinct advantages in visualization and repeatability [15]. However, most current obstetric VR modules focus on fragmented, single-skill training—such as managing labor positions [16] and practicing breastfeeding techniques [17]—rather than integrating prenatal assessment, intrapartum management, postpartum care, and complication response into a unified, continuous clinical scenario. This fragmented approach hinders the development of clinical thinking across the entire perinatal continuum and may ultimately compromise students’ clinical adaptability and professional competence [15,18]. Therefore, the development of a contextually progressive VR platform centered on the continuity of obstetric care is essential for optimizing existing systems and enhancing students’ clinical thinking.

Labor is a normal physiological process, and its health care and research should be reconstructed from a perspective that is opposed to pathology [19]. Salutogenesis conceptualizes health as a dynamic continuum that ranges from severe illness to optimal well-being. This model emphasizes mobilizing both existing and potential resources—including biological, psychological, cultural, and social factors—to promote movement toward the healthier end of the wellness spectrum [20]. On the basis of this theoretical framework, our team collaborated with a Chinese technology company to develop a Nonimmersive Virtual Reality System for Clinical Thinking in Obstetric Nursing (NIVRSCTON). It is an interactive digital platform designed to cultivate and enhance obstetric nursing students’ clinical thinking ability and professional competence through simulated obstetric scenarios. It was designed to address 2 critical limitations of existing obstetric VR modules, such as clinical fragmentation and the absence of contextual progression. By simulating continuous labor support, which ranges from normal labor and complication management to postpartum care for laboring women and newborns, the system helps bridge the gap between isolated skills training and the cultivation of integrated clinical thinking.

The NIVRSCTON is reproducible, vivid, interactive, and engaging. It integrates a multidimensional perspective on labor, combining diverse resources to establish a robust support system aimed at improving the birth experience and ensuring the safety of both laboring women and newborns. Rooted in authentic clinical cases, the system allows students to assess and predict labor progression, make evidence-based decisions, and cultivate the clinical competencies necessary to facilitate positive birth outcomes.

Upon completion of the NIVRSCTON, the research team integrated it into the practical teaching of obstetrics and gynecology nursing based on Kolb’s experiential learning theory [21]. Kolb’s experiential learning theory is a scientific pedagogical theory that consists of 4 stages. Learners can follow the cyclic sequence of “concrete experience—reflective observation—abstract conceptualization—active experimentation” for learning, or may randomly enter any stage of learning. Therefore, during the practical teaching of the obstetrics and gynecology nursing course, nursing students acquired foundational course knowledge and were subsequently required to access the online NIVRSCTON platform. Through this platform, they experienced and implemented continuous childbirth care in nonimmersive VR scenarios with reflective observation. Following the online training, students were expected to conduct holistic reflections based on the Bass model [22]. This process aimed to consolidate their theoretical knowledge, strengthen their practical competencies, and cultivate clinical thinking. This holistic reflection also serves as a critical foundation for students’ subsequent clinical visits and internships, better preparing them to encounter real-world clinical scenarios effectively.

Based on the aforementioned teaching design, we conducted a pre-post design study across 4 universities in China involving 88 third-year undergraduate nursing students to evaluate the effect of NIVRSCTON training. This study aimed to (1) evaluate the ability of NIVRSCTON to cultivate obstetric clinical thinking among nursing students, (2) measure its influence on enhancing students’ comprehensive competencies, and (3) explore students’ perceptions and evaluations of using NIVRSCTON during obstetric nursing training.

## Methods

### Study Design

This study used a single-group pre-post design and an explanatory sequential mixed methods approach, with no separate control group included. Using methodological triangulation, this study first used a quantitative research method to assess changes in students’ clinical thinking after they used the NIVRSCTON training system and evaluated its effectiveness. Qualitative data were then collected through reflective journals to gain deeper insights into students’ experiences and perceptions during the training process. Finally, during the interpretation phase, quantitative and qualitative findings were integrated to evaluate the convergence or divergence of the data. This study followed the Good Reporting of a Mixed Methods Study (GRAMMS) guidelines [23] for mixed methods reporting; the full 6-item checklist is shown in Multimedia Appendix 1.

### Study Process

**Figure 1:**
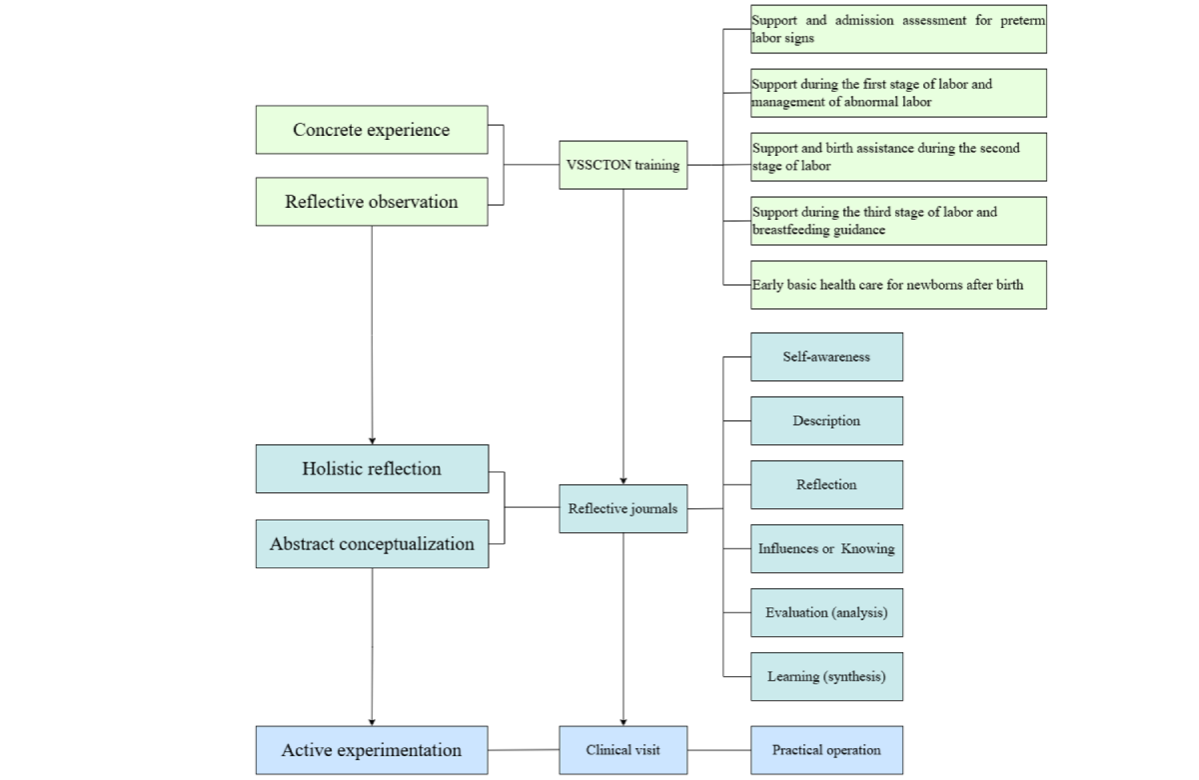
Online training using the Nonimmersive Virtual Reality System for Clinical Thinking in Obstetric Nursing (NIVRSCTON) for nursing students, based on Kolb’s experiential learning theory.

This research team conducted the NIVRSCTON training based on Kolb’s experiential learning theory online for third-year nursing students who were enrolled in an obstetrics and gynecological nursing course (Figure 1).

### Concrete Experience and Reflective Observation

After the students finished the theoretical learning section of obstetrics and gynecology nursing, online training using the NIVRSCTON was delivered via Tencent Meeting (Chinese Tencent). The teacher explained the instructions and showed how to use the system online. When students accessed the NIVRSCTON, they experienced a continuous process of childbirth care. Through interactive scenarios involving the laboring woman, doctors, and other providers, they conducted comprehensive assessments of the woman, formulated nursing diagnoses, and provided obstetric support and health education. If an abnormal situation arose during the training, students were required to analyze the potential causes, identify key contributing factors, and make appropriate clinical decisions. Following the birth, students also provided essential early newborn care. In every scenario, students were required to consciously use reflective observation to generate valuable insights and knowledge that guided their subsequent actions. They had 1 hour and 35 minutes to complete the training, and each scenario had a time limit with irreversible progress. Multimedia Appendix 2 shows the specific scene content and screenshots from several scenes.

### Holistic Reflection and New Concept Formation

After the online training using the NIVRSCTON, students wrote a reflective journal within 1 week and submitted it via WeChat (Chinese Tencent). Reflective journals are grounded in the Bass model [22]. Students needed to identify their initial thoughts or biases during training. Then, they objectively described the experience or content, followed by a deeper reflection on their personal and professional significance. Next, they analyzed the impact on their understanding and evaluated the underlying mechanisms. Finally, they integrated these insights into new obstetric knowledge or skills, which informed their self-awareness for future clinical practice, creating a continuous cycle of growth.

### Active Experimentation

Equipped with the knowledge, skills, and behaviors developed in the previous stages, the students proceeded to the next phase of their training—practical clinical visits.

### Study Population

This training was hosted by the Nursing Virtual Teaching Hub in Coastal Area (NVTHCA), co-organized by Hainan Medical University and Hangzhou Normal University, and supported by a Chinese technology company. The Virtual Teaching and Research Office is a national initiative launched under China’s higher education digital transformation agenda. It was designed to establish nationwide or regional university-level teaching and research networks that leverage digital platform technologies to enable the co-construction and sharing of educational resources across time and space. The NVTHCA, approved in 2022 as a part of the second batch of national projects by the Ministry of Education, is led by Hainan Medical University and jointly established by 13 coastal universities’ schools of nursing. In September 2023, undergraduate students from 4 co-construction universities (in Hainan, Guangdong, Guangxi, and Fujian provinces) were recruited. Inclusion criteria were as follows: (1) willing to participate voluntarily, (2) completion of chapters on normal and abnormal labor in the obstetrics and gynecology nursing course, (3) basic computer knowledge, and (4) no previous experience with VR teaching. Exclusion criteria were as follows: (1) previous completion of the obstetrics and gynecology nursing course or being held back a grade, and (2) having changed their major.

### Data Collection

A general information questionnaire and the Clinical Thinking Ability Evaluation Scale (CTAES) were sent to the students through WeChat before the training. After the training, the researcher used the CTAES and the Evaluation Instrument for Virtual Reality System (EIVRS) to collect the data (Multimedia Appendix 3). Qualitative data were collected from reflective journals written by the students.

### General Information Questionnaire

A general information questionnaire, which included age, sex, grade, case study experience, and VR teaching or learning experience, was designed based on a literature review.

### Clinical Thinking Ability Evaluation Scale

This scale was developed by Song [9] to assess the clinical thinking ability of medical students. It has 3 dimensions—systematic thinking, critical thinking, and evidence-based thinking—and includes 24 items. Each item was assigned on a 5-point Likert scale ranging from 1 (very poor) to 5 (excellent). The raw total of 120 points was converted into a percentage for final reporting. Scores from 80 to 100 indicate excellent clinical thinking; scores from 60 to 79 indicate good ability; scores from 40 to 59 indicate average ability; scores from 20 to 39 suggest poor ability; and scores from 0 to 19 reflect very poor clinical thinking. The test-retest reliability score of this scale is 0.84, while the Cronbach *α* score is 0.91 [9].

### Evaluation Instrument for VR System

The EIVRS was developed by Chen [24] to assess the performance of VR software in surgical nursing education. It has 15 items, and the overall score ranges from 0 to 10. It contains 4 domains—interface design (items 1-3; maximum score 1.5), technical performance (items 4-5; maximum score 1.5), learning content (items 6-12; maximum score 3), and learning function (items 13-15; maximum score 4). Each domain has weighted subitems. The following are the subitems along with their weights and coefficients: aesthetic style (5/1.25), media design (6/1.50), link design (4/1.00), navigation (6/1.50), interactivity (9/2.25), instructional material (18/4.50), resource quality (12/3.00), module division (8/2.00), learning assessment (12/3.00), and learning outcome (20/5.00). Subitem scores are calculated as (raw score × coefficient)/items per subitem; the composite score equals the actual total/10 (range 0-1), with >0.85 indicating excellent, 0.70-0.84 indicating good, 0.60-0.69 indicating moderate, and <0.60 indicating poor performance. The content validity index of the EIVRS is 0.80 [25].

### Reflective Journals

Reflection is a practice that enhances students’ self-awareness and facilitates an in-depth examination of their learning processes. Through reflective activities, students are enabled to better navigate their learning pathways, unlock their potential, and develop critical thinking. The reflective journal serves as a key instrument for fostering such reflection. It is a written medium through which students record events, experiences, thoughts, and insights and subsequently engage in active review, analysis, and synthesis. This process functions as a form of introspective dialog, promoting deeper thinking and the consolidation of experiential knowledge [26]. Students were required to write a reflective journal based on the Bass model proposed by Australian scholar Bass [22]. This reflection model consists of 6 components—self-awareness, description, reflection, impact or cognition, evaluation (analysis), and learning (integration)—which are sequentially connected to form a circular structure. Its core objective is to construct a learner-centered learning environment and support learners in conducting systematic and holistic reflection activities throughout the entire learning cycle.

After all students completed the NIVRSCTON training, the research team immediately conducted a 30-minute online reflective journal guidance session via Tencent Meeting. During this session, the team explained the purpose and requirements for writing reflective journals based on the Bass Model of Holistic Reflection. The researchers explained each of the 6 components of the Bass model (Multimedia Appendix 4) and specified the formatting guidelines and submission deadline (within 1 week after training). Students were able to consult the research team at any time via a WeChat group if they had questions during the writing process.

### Data Analysis

The quantitative data were analyzed using IBM SPSS Statistics (version 22.0). Categorical variables were described as frequencies and percentages, and continuous, normally distributed variables were described as means (SD). Paired, 2-tailed t tests were used to assess within-group changes, and a *P* value of <.05 was considered statistically significant. Effect sizes were calculated using G*Power (Heinrich-Heine-Universität Düsseldorf and Franz et al [27]), and their interpretation followed conventional thresholds: small (*d*=0.2), medium (*d*=0.5), and large (*d*≥0.8) [28].

Qualitative data were analyzed via NVivo (version 12) software. To protect students’ personal privacy, their names in the reflective journals were replaced by “S” followed by a serial number. The researchers used an inductive content analysis method to extract themes [29]. This process included four steps: (1) reading the reflective journals carefully to gain an overall sense of their content, (2) identifying important sentences in the reflective journals, (3) defining the classification system of themes and subthemes, and (4) extracting representative quotations from the reflective journals. Two researchers independently conducted the entire procedure, repeatedly reading the reflective journals until no new themes emerged. Their individual code sets were subsequently merged and discussed until consensus on meaningful codes and themes was achieved. A third team member with qualitative expertise resolved ambiguities to ensure data validity.

Qualitative data saturation was determined using thematic saturation [30]. Two researchers independently analyzed all the reflective journals in the order of student submission, recording each new theme as it first appeared. The qualitative analysis initially reached thematic saturation after 45 journals were reviewed. The researchers then merged their thematic findings. Following theme integration, the researchers continued examining the remaining journals. Any emerging themes during this phase were discussed and incorporated upon mutual agreement. A third researcher with qualitative expertise resolved discrepancies to ensure data validity.

### Ethical Considerations

This study was approved by the ethics committee of Hangzhou Normal University (approval no 2023106) and conducted in accordance with the Declaration of Helsinki. Written informed consent was obtained from the participants before the training. All the data were anonymized using unique alphanumeric codes and kept strictly confidential throughout the study. Students could withdraw at any time without penalty, and no compensation was provided.

## Results

### Students’ Characteristics

A total of 88 third-year nursing undergraduates who were about to commence clinical placement were recruited during their second semester. All the students were female, with a mean age of 20.28 (SD 0.64) years. None of them had previously used VR for training, and 56% had previous experience in case-based learning.

### Changes in Students’ Clinical Thinking

Students demonstrated significant improvements across all measured domains after training. Overall clinical thinking increased from 49.08 (SD 11.30) to 80.50 (SD 11.01; *t*_87_=−18.76; Cohen *d*=−2.82, 95% CI −34.74 to −28.08). Critical thinking increased from 13.98 (SD 3.76) to 24.77 (SD 3.11; *t*_87_=−20.37; Cohen *d*=−3.13, 95% CI −11.85 to −9.74). Systems thinking increased from 26.82 (SD 6.40) to 44.51 (SD 6.24; *t*_87_=−19.18; Cohen *d*=−2.80, 95% CI −19.53 to −15.86). Evidence-based thinking increased from 18.10 (SD 4.40) to 27.31 (SD 4.61; *t*_87_=−13.42; Cohen *d*=−2.04, 95% CI −10.57 to −7.84; Table 1).

**Table 1:** Changes in students’ overall clinical thinking, critical thinking, systems thinking, and evidence-based thinking before and after the Nonimmersive Virtual Reality System for Clinical Thinking in Obstetric Nursing (NIVRSCTON) training (N=88).

Primary indicator	Before training, mean (SD)	After training, mean (SD)	*t* test (*df)*	*P* value	Cohen *d* (95% CI)
System thinking	26.82 (6.40)	44.51 (6.24)	–19.18 (87)	<.001	–2.80 (–19.53 to –15.86)
Critical thinking	13.98 (3.76)	24.77 (3.11)	–20.37 (87)	<.001	–3.13 (–11.85 to –9.74)
Evidence-based thinking	18.10 (4.40)	27.31 (4.61)	–13.42 (87)	<.001	–2.04 (–10.57 to –7.84）
Overall clinical thinking	49.08 (11.30)	80.50 (11.01)	–18.76 (87)	<.001	–2.82 (–34.74 to –28.08)

### Student Evaluation of the NIVRSCTON Performance

The students rated the NIVRSCTON as follows: interface design (mean 0.82, SD 0.15), technical performance (mean 0.82, SD 0.15), learning content (mean 0.83, SD 0.14), learning function (mean 0.85, SD 0.15), and overall evaluation (mean 0.82, SD 0.15). The domains were ranked from highest to lowest as follows: learning function, learning content, interface design, and technical performance (Table 2).

**Table 2:** Post training evaluation of the Nonimmersive Virtual Reality System for Clinical Thinking in Obstetric Nursing (NIVRSCTON) by students (N=88).

Primary indicator	Weight (score)	Score, mean (SD)	Rating	Ranking
Interface design	1.5	0.82 (0.15)	Good	3
Technical performance	1.5	0.82 (0.15)	Good	3
Learning content	3	0.83 (0.14)	Good	2
Learning function	4	0.85 (0.15)	Excellent	1
Overall evaluation	—^a^	0.82 (0.15)	Good	—

^a^Not available.

### Results of Students’ Reflective Journals

Three major themes were abstracted from the reflective journals: (1) reflection on practical actions, (2) reflection on practical ability, and (3) reflection on multidimensional factors influencing training. The 3 themes and their 12 subthemes, with some examples, are provided below. Additional results are provided in Multimedia Appendix 5.

#### Theme 1: Reflection on Practical Actions

##### Overview

The students highlighted the importance of maintaining composure while providing support to ensure continuous assessment and guidance throughout labor. They also emphasized the value of providing timely, humanistic care and building a collaborative partnership with women. The training reinforced their dedication to professional integrity, emphasizing the need for ethical vigilance even in VR contexts.

##### Subtheme 1.1 Maintaining Calm

From the reflective journals, some students noted that the training cultivated their ability to maintain calm in highly stressful situations. In obstetrics, unexpected circumstances may arise at any time; maintaining composure is not only a demonstration of personal professional quality but also a crucial ability to ensure the safety of the laboring woman and newborn and to respond efficiently to emergencies.

Paying attention to one’s own emotions and state to ensure remaining calm and focused is also key to giving confidence to the laboring woman and her family.

S8

##### Subtheme 1.2 Assessment and Support Throughout Labor

Within the NIVRSCTON environment, students gained a profound understanding that assessment and intervention must be integral throughout the entire labor process. They thought it was essential to make accurate judgments and implement targeted nursing interventions for both the laboring woman and the newborn.

We have performed a series of comprehensive nursing assessments, diagnoses, health education, and support for the laboring woman. These measures are aimed at ensuring the safety of both the laboring woman and newborn.

S1

##### Subtheme 1.3 Humanistic Care

During the NIVRSCTON training, students themselves understand the physical, mental, and emotional changes experienced by women throughout the childbirth process, thereby enabling them to provide targeted support. Some students reported that this training fostered a profound understanding of the critical role of humanistic care in obstetric nursing. This care not only alleviates the anxiety and fear of women but also enhances their confidence and willingness to cooperate with nurses during childbirth.

When experiencing pain, the laboring woman receives my empathetic support, along with evidence-based labor analgesia strategies to effectively relieve her discomfort and enhance her confidence in the birthing process.

S9

##### Subtheme 1.4 Establishing Partnerships

Students recognized that building a trusting partnership with women facilitates effective communication, mutual understanding, and emotional support, thereby enabling them to better navigate challenges during childbirth.

Foster a strong partnership with the woman and her family to ensure that the woman receives a comprehensive understanding and support throughout the delivery process.

S8

##### Subtheme 1.5 Avoiding Medical Risks

During the NIVRSCTON training, students recognized that this type of training can help reduce clinical risks associated with real obstetric settings.

Omitting any step in a VR scenario may not result in significant consequences. However, it can help me avoid medical risks in real obstetric environments.... I know in real-world clinical practice, such seemingly minor errors can have devastating, irreversible consequences for both the mother and her newborn.

S41

#### Theme 2: Reflection on Practice Ability

##### Overview

Students reported that the training served as an effective bridge between theoretical knowledge and clinical practice, enhancing their clinical thinking, reinforcing their professional identity, and promoting a collaborative mindset.

##### Subtheme 2.1 Promoting the Integration of Theoretical Knowledge With Practical Application

Within the VR environment, students were able to coherently carry out nursing care procedures, thereby facilitating the flexible application of theoretical knowledge to clinical practice and effectively fostering the integration of knowledge with practical skills.

In the dynamic and continuous process support, I need to promptly identify problems and provide assistance. Applying the knowledge, I have learned flexibly to the practical training helps to make up for the shortcomings of traditional training and enhances my abilities.

S32

##### Subtheme 2.2 Enhancing Adaptability in Training

Students reported that they were required to identify problems, analyze causes, and take effective measures during both normal and abnormal deliveries within the prescribed time, which helped them enhance their adaptability and become accustomed to clinical practice in advance.

“The system covers the abnormal delivery process. I need to identify and handle the problems within a limited time, which helps to exercise my adaptability and clinical thinking.”

S2

##### Subtheme 2.3 Strengthening Evidence-Based Thinking

In a highly simulated clinical environment with dynamically evolving birthing scenarios, students were encouraged to move beyond the mechanical repetition of procedural steps and instead engage in critical analysis of underlying causes. Some students noted that this approach effectively enhanced their evidence-based thinking.

“In the classroom, we learned that after the fetus is delivered, assistance is needed for the repositioning of the fetal head and external rotation. However, after this training session, I learned from the latest guidelines that there are certain points regarding not rushing to assist with the repositioning of the fetus, external rotation, and delivery of the shoulders.”

S6

##### Subtheme 2.4 Fostering Professional Identity

When students successfully resolved clinical problems using evidence-based approaches, they reported increased self-efficacy and a clearer sense of their future professional value and role.

The training enabled me to grasp critical concepts, such as the recommendation in the latest clinical guidelines, to avoid hastening shoulder delivery. This experiential learning has fundamentally reshaped my clinical understanding and is expected to yield lasting positive effects on my future academic pursuits and professional growth.

S6

##### Subtheme 2.5 Facilitating Team Collaboration

When students encountered clinical challenges, they collaborated with simulated doctors, nurses, and other members of the health care team to jointly develop solutions within the NIVRSCTON. This process helped them foster a strong sense of teamwork.

I need to maintain close communication and collaboration with members of the medical team... Ensure the safety of both the laboring woman and the newborn.

S5

#### Theme 3: Reflection on the Multidimensional Factors Influencing Practice

##### Overview

Factors influencing students’ learning and actions in the VR training include both internal and external elements. Internal factors such as beliefs, values, a sense of responsibility, and knowledge motivate students to engage in practical actions. External factors, such as resources, policies, and the sociocultural environment, play a role in shaping students’ decisions and actions. Moreover, students pointed out certain system configuration deficiencies, offering directions for future improvements.

##### Subtheme 3.1 Internal Factors

The internal factors inherent to students propel them to translate the knowledge they have acquired into practical applications and enable them to make informed decisions in the context of labor. Such influences stem from a variety of sources, including their knowledge, skills, psychological attributes, personality traits, interests, emotions, beliefs, sense of responsibility, and core values.

Scientific and authoritative guidelines, textbooks, and skills are the primary factors influencing my clinical decision-making and actions.

S2

Pressure and a sense of responsibility will compel me to maintain a calm demeanor, systematically analyze the circumstances surrounding the laboring woman, and deliver appropriate midwifery support.

S4

##### Subtheme 3.2 External Factors

External factors include the degree of authenticity of the VR training environment, unfamiliarity with system operation, the sense of urgency created by the countdown, the lack of humanity in the system, and the need to optimize the system’s performance.

The system interface encompasses a variety of functions and content. Occasionally, I inadvertently select information that is not pertinent to the current stage of assessment... Furthermore... It would be advantageous to incorporate both prompt subtitles and audio features.

S7

The countdown within the system induced a significant level of tension in me during the training.

S20

Unknown uncertainties, the social and cultural environment, and the attitudes and behaviors of the laboring woman and her family members also influenced the students’ decision-making in the NIVRSCTON training.

The availability of hospital resources, policies, medical equipment, and other factors will influence my decision-making process.

S5

### Integrated Findings

The quantitative and qualitative findings in this study were mutually validated and complemented. A detailed integration is provided in Multimedia Appendix 6. After the training, the students significantly improved their system thinking, critical thinking, and evidence-based thinking. The training also promoted practical behaviors related to assessment and guidance, enhanced the integration of theoretical knowledge with practical application, and strengthened their ability to adapt to changing situations.

In addition, the training effectively improved students’ practical competence in remaining calm, providing humanistic care, building partnerships, and avoiding medical risks. It also facilitated team collaboration and fostered professional identity.

The students rated the system application positively. The scores, in descending order, were as follows: learning function, learning content, interface design, and technical performance. The relatively lower ratings for interface design and technical performance were influenced mainly by external factors such as the highly simulated VR environment, situational uncertainties, and the countdown function within the system.

## Discussion

### Principal Findings

This study used a mixed method design to assess the effects of the NIVRSCTON on obstetric training for third-year undergraduate nursing students. The implementation of this VR training in 4 regions constitutes a novel contribution to the digital transformation of higher education in China. The quantitative results revealed that the NIVRSCTON system significantly enhanced students’ clinical thinking (systems thinking, critical thinking, and evidence-based thinking) and professional competence. Students rated the system’s learning functions, learning content, interface design, and technical performance highly. Qualitative findings revealed that the training enhanced students’ ability to apply clinical skills in simulated scenarios and refined their overall practical competence. Multidimensional factors, including internal factors (knowledge, skills, emotions, beliefs, sense of responsibility, and values) and external factors (training environment, countdown timer, and system technical performance), influenced their training. Therefore, implementing NIVRSCTON training can effectively enhance students’ clinical thinking and comprehensive competencies while addressing the inherent fragmentation and absence of progressive contextualization in traditional obstetric nursing practice.

Systems thinking emphasizes moving beyond surface-level phenomena to uncover the root causes of complex issues, understand interconnections among components, identify leverage points, and ultimately develop effective and sustainable solutions [31]. Our findings indicate that the significant improvement in students’ systems thinking abilities following NIVRSCTON training can be attributed to the integration of the salutogenesis model and Kolb’s experiential learning theory into its development and application. The NIVRSCTON replicates a continuous care process from the early signs of labor through the first 2 hours post partum, integrating the identification and management of obstetric complications such as abnormal fetal heart rate, premature rupture of membranes, and fetal malposition. When assessing labor progress and providing nursing care, students are required to engage in comprehensive reflection and analysis of information through the application of experiential learning skills. They identified underlying causes of obstetric abnormalities, recognized critical factors, and made appropriate clinical decisions. Furthermore, reflective journals from students indicated that NIVRSCTON training enabled them to perform continuous, systematic evaluations and provide support for women throughout the simulated labor process. This experience enhanced their ability to promptly identify nursing issues, reinforced their theoretical knowledge, enhanced their practical behaviors, and addressed the limitations of realistic VR training and clinical practice in the real obstetric environment [14]. Thus, the NIVRSCTON training not only effectively developed students’ systems thinking but also specifically addressed the gaps in the coverage of real-world obstetric nursing VR scenarios, providing a valuable reference for virtual reality–based nursing education.

Critical thinking refers to a higher-order cognitive process that enables students to dynamically integrate previous knowledge and experiential learning to analyze, reason, and form sound clinical judgments within complex clinical scenarios [9]. The students’ enhanced critical thinking was fostered by NIVRSCTON training based on Kolb’s theory. These scenarios required students to dynamically integrate parturient assessment, effective communication, and theoretical knowledge under time pressure, progressing from reflective observation to clinical decision-making. Unlike traditional training models based on unidirectional knowledge transmission, NIVRSCTON training uses interactive, dynamically evolving obstetric scenarios to foster students’ engagement and enhance critical thinking skills. These findings are similar to those reported by Yeo et al [32], who developed a VR for pediatric pneumonia care covering the entire process from admission to discharge. Furthermore, the results of this study revealed that when critical thinking was applied to successfully manage obstetric emergencies, students reported enhanced adaptive competence and better preparedness for future clinical practice. Therefore, in addition to cultivating students’ critical thinking skills, NIVRSCTON training also contributes to enhancing nursing students’ adaptability to clinical practice and overall competence.

Evidence-based thinking involves the critical appraisal, logical synthesis, and application of valid evidence to guide clinical thinking and support sound decision-making in practice [33]. In this study, there was a significant improvement in students’ evidence-based thinking following NIVRSCTON training. We designed case scenarios and test items in accordance with the latest Chinese guidelines on normal labor and expert consensus on early neonatal care. This process adhered to established guidelines for VR-based education, which mandate that nursing scenarios be rigorously grounded in evidence-based practice [34]. This training enabled students to promptly access and apply valid evidence within VR scenarios while facilitating dialectical analysis and logical reasoning. For instance, several students reported receiving guidance during NIVRSCTON training, stating that “there is no need to hastily assist with fetal restitution, external rotation, or shoulder delivery” during labor. This approach differed significantly from traditional labor techniques taught in classrooms and during clinical visits. This experience prompted students to critically evaluate conventional practices, engage in analytical and logical reasoning, and ultimately arrive at evidence-based clinical decisions. This outcome is similar to the findings of Ayibulati et al [35], who applied a VR platform to Tuina therapy education guided by the principles of evidence-based medicine. Therefore, cultivating evidence-based thinking through NIVRSCTON training has demonstrated potential as both a feasible and effective approach.

Traditional clinical training in obstetric nursing often overlooks the development of students’ affective and attitudinal competencies [36]. However, in NIVRSCTON training, which was based on Kolb’s experiential learning theory, students reported that their emotional responses, attitudes, and cognitive understanding were enhanced. Students underwent a process of “concrete experience–reflective observation–abstract conceptualization” within NIVRSCTON training [21,22]. When encountering emergencies in the system, students learned to remain calm and respond effectively to ensure the safety of laboring women and newborns. Moreover, when in the VR environment and using reflective observation, students could understand the feelings of women and recognize the importance of providing humanistic care, building trust, and establishing partnerships in maternity care. A previous study also found that VR training provides students with more opportunities to enhance their practical skills while fostering a rigorous attitude and a sense of professional integrity and ethical self-discipline [16].

In their reflection journals, students noted that their VR experience and simulated teamwork not only helped them master multidisciplinary collaboration models but also strengthened their identification with professional values and missions. These findings align with existing research: Edgar et al [37] reported that VR enhances students’ experience and sense of identity with their professional roles, whereas Neher et al [38] confirmed that interdisciplinary VR collaboration effectively develops teamwork skills. Furthermore, guided by Kolb’s model [21,22], this study implemented “holistic reflection and abstract conceptualization” and revealed that students’ ability to translate knowledge into action and make calm decisions during dynamic childbirth scenarios stemmed from internal factors such as knowledge, skills, and a sense of responsibility. These findings are consistent with the research by Lee et al [39], who also demonstrated that students with solid knowledge and high responsibility exhibited greater composure and confidence in VR, such as intravenous injection training. Thus, NIVRSCTON training enhances students’ professional competence by strengthening their practical behaviors and abilities, thereby facilitating the transformation of internal qualities into concrete actions. In addition, some students reported that the VR provided a “zero-risk” trial environment before clinical practice, where erroneous decisions would not cause actual harm. This significantly increased their confidence when they entered real obstetric nursing scenarios. This finding indicates that NIVRSCTON training effectively promotes the transition from experiential learning to “active practice.”

Students generally provided positive feedback on the effectiveness of the NIVRSCTON system. Dimension ratings in descending order were as follows: learning function, learning content, interface design, and technical performance. The system includes 6 modules along with 5 logically structured and progressively challenging clinical scenarios, and its functions and content received high evaluations. Although the interface design intuitively visualized the childbirth process, some students reported that cluttered interface elements occasionally led to unintended interactions or distractions. Despite standardized operational instruction before the training, some students still demonstrated operational unfamiliarity and nervousness. Research has indicated that visual appeal and usability are crucial for sustaining learning motivation [40,41]. The technical performance dimension received relatively lower ratings. Students suggested adding voice prompts and optimizing the text layout to reduce visual load. Studies have shown that voice guidance can enhance situational immersion [32]. Furthermore, students revealed that the high-fidelity environment, situational uncertainties, and countdown settings were prone to inducing anxiety, thereby affecting clinical decision-making. This aligns with findings that initial exposure to VR systems often triggers students’ stress responses due to unfamiliarity [42]. Scholars recommend implementing progressive scenario exposure, real-time feedback, and stress regulation mechanisms to improve the user experience [43]. Future iterations of the NIVRSCTON system should prioritize interface refinement and technical enhancements to address these areas identified for improvement.

### Limitations

There were several limitations to this study. First, the CTAES and EIVRS were developed based on reliability and validity testing of their application for assessing clinical thinking and VR performance among Chinese medical students. These 2 assessment tools have been widely used in educational reform studies in surgical, obstetric, and medical nursing in China. However, their reliability and validity have not yet been specifically examined within the population of Chinese nursing students, which may slightly compromise the reliability of the findings. As this study supplemented the evaluation of nursing students’ obstetric clinical thinking and the performance of NIVRSCTON by analyzing qualitative data derived from reflective journals, it may partially compensate for the absence of population-specific validation of the CTAES and EIVRS. Second, all the participants were third-year female nursing students. The absence of male participants may be caused by the extremely low proportion of male nursing students in China and their lower interest in obstetric and gynecological nursing courses. Consequently, the sex homogeneity of the sample may limit the generalizability of the findings to a broader population. We plan to adopt strategies such as emphasizing the husband’s role in the childbirth process to engage male students, which may enhance the generalizability of this training in future research. Third, only one training session was conducted since the development of the VR system. It is necessary to address shortcomings such as the lack of real-time feedback, the absence of voice prompts, and anxiety induced by time-limited tasks to enhance its usability and instructional effectiveness.

### Conclusions

Grounded in the salutogenesis theory and Kolb’s experiential learning theory, NIVRSCTON training demonstrated meaningful effectiveness among nursing students by significantly enhancing clinical thinking (systems thinking, critical thinking, and evidence-based thinking) and professional competence. It may serve as a valuable auxiliary tool for fostering clinical thinking and facilitating the transition to clinical practice among nursing students. Moreover, this study used a Chinese virtual teaching and research platform to enable cross-regional and cross-institutional application of high-quality educational resources under the principle of “cocreation and sharing.” This approach may promote equity and accessibility in nursing education by alleviating disparities in educational resource allocation. It may also provide an innovative pathway and case study for digital nursing education. Finally, future work should focus on further system optimization and exploring optimal instructional designs while expanding its application to other academic fields to verify its long-term impact and cross-disciplinary applicability.

## Appendix

Multimedia Appendix 1 This study adheres to the Good Reporting of a Mixed Methods Study (GRAMMS) guidelines for mixed methods reporting.

Multimedia Appendix 2 The Virtual Simulation System for Clinical Thinking in Obstetric Nursing (VSSCTON) includes 5 specific scenarios and screenshots from multiple scenarios.

Multimedia Appendix 3 The specific content of the quantitative data collection process.

Multimedia Appendix 4 Writing requirements for reflective journals based on the Bass model.

Multimedia Appendix 5 Summary table of qualitative themes with additional representative quotes.

Multimedia Appendix 6 Integration of quantitative and qualitative research results.

## Abbreviations

CTAES: Clinical Thinking Ability Evaluation Scale
EIVRS: Evaluation Instrument for Virtual Reality System
GRAMMS: Good Reporting of a Mixed Methods Study
NIVRSCTON: Nonimmersive Virtual Reality System for Clinical Thinking in Obstetric Nursing
NVTHCA: Nursing Virtual Teaching Hub in Coastal Area
VR: virtual reality

## Data Availability

The datasets generated or analyzed during this study are not publicly available due to students’ privacy concerns, but are available from the corresponding author on reasonable request.
