# Supplementary material for: Use of a Nonimmersive Virtual Reality System for Clinical Thinking in Obstetric Nursing Education: Mixed Methods Study
Source: J Med Internet Res. 2025 Nov 24;27:e80951. doi: 10.2196/80951 (PMC12686860; doi:10.2196/80951)
Supplement: Multimedia Appendix 1 [file jmir_v27i1e80951_app1.docx]

**Good Reporting of a Mixed Methods Study (GRAMMS)**

This study follows the GRAMMS guideline developed by O’Cathain A in 2008, which comprises 6 core reporting items.

| **Guideline** | **Execute** | **Section: page** |
| --- | --- | --- |
| Describe the justification for using a mixed methods approach to the research question. | √ | In the methods section: pg. 3 |
| Describe the design in terms of the purpose, priority and sequence of methods. | √ | In the methods section: pg. 3-6 |
| Describe each method in terms of sampling, data collection and analysis. | √ | In the methods section: pg. 3-6 |
| Describe where integration has occurred, how it has occurred and who has participated in it. | √ | In the methods section: pg. 3-5; 9 |
| Describe any limitation of one method associated with the present of the other method. | √ | In the methods section: pg. 3 |
| Describe any insights gained from mixing or integrating methods. | √ | In the discussion section: pg. 9-11 |

**References**

O’Cathain A, Murphy E, Nicholl J. The quality of mixed methods studies in health services research. J Health Serv Res Policy, 2008, 13: 92-98. [doi: 10.1258/jhsrp.2007.007074] [Medline:18416914]
