# Supplementary material for: Use of a Nonimmersive Virtual Reality System for Clinical Thinking in Obstetric Nursing Education: Mixed Methods Study
Source: J Med Internet Res. 2025 Nov 24;27:e80951. doi: 10.2196/80951 (PMC12686860; doi:10.2196/80951)

Based on salutogenesis theory, the Nonimmersive Virtual Reality System for Clinical Thinking in Obstetric Nursing (NIVRSCTON) was developed with 5 scenarios modules covering prodromal labor, postpartum care, and early neonatal basic health care.

| Scenario | Content | Time  (minutes) |
| --- | --- | --- |
| Scenario 1: support and admission assessment for preterm labor signs | 1. Correctly distinguishing pre-labor signs from true labor.  2. Judging the timing of hospital admission.  3. Management of labor pain.  4. Self-monitoring by laboring woman. | 10 |
| Scenario 2: support during the first stage of labor and management of abnormal labor | 1. Admission and initial assessment.  2. Recognition and management of uterine atony.  3. Recognition and management of abnormal fetal heart rate.  4. Management of premature rupture of membranes.  5. Management of the urge to defecate.  6. Recognition and management of abnormal fetal position.  7. Management of labor pain. | 35 |
| Scenario 3: support and birth assistance during the second stage of labor | 1. Evaluation during the second stage of labor.  (1) Evaluation of the basic condition of the laboring woman.  (2) Evaluation of the progress of labor.  2. Midwifery care during the second stage of labor.  (1) Guidance on exertion at different stages of labor.  (2) Preparation for birthing.  (3) Timing for going to the birthing table.  (4) Birthing assistance. | 20 |
| Scenario 4: support during the third stage of labor and breastfeeding guidance | 1. Evaluation and midwifery support during the third stage of labor and within 2 hours postpartum.  2. Correct identification of signs of placental separation.  3. Breastfeeding guidance.  4. Health education on perinatal-related knowledge. | 15 |
| Scenario 5: early basic health care for newborn after birth | 1. Immediate early essential care for the newborn after birth.  2. Early essential care for the newborn within 3 minutes after birth.  3. Early essential care for the newborn from 3 minutes to 90 minutes after birth.  4. Early essential care for the newborn after 90 minutes of birth. | 15 |

**Figure S1.** The dialogue interface between the virtual obstetric nurse and the virtual laboring woman in the system.


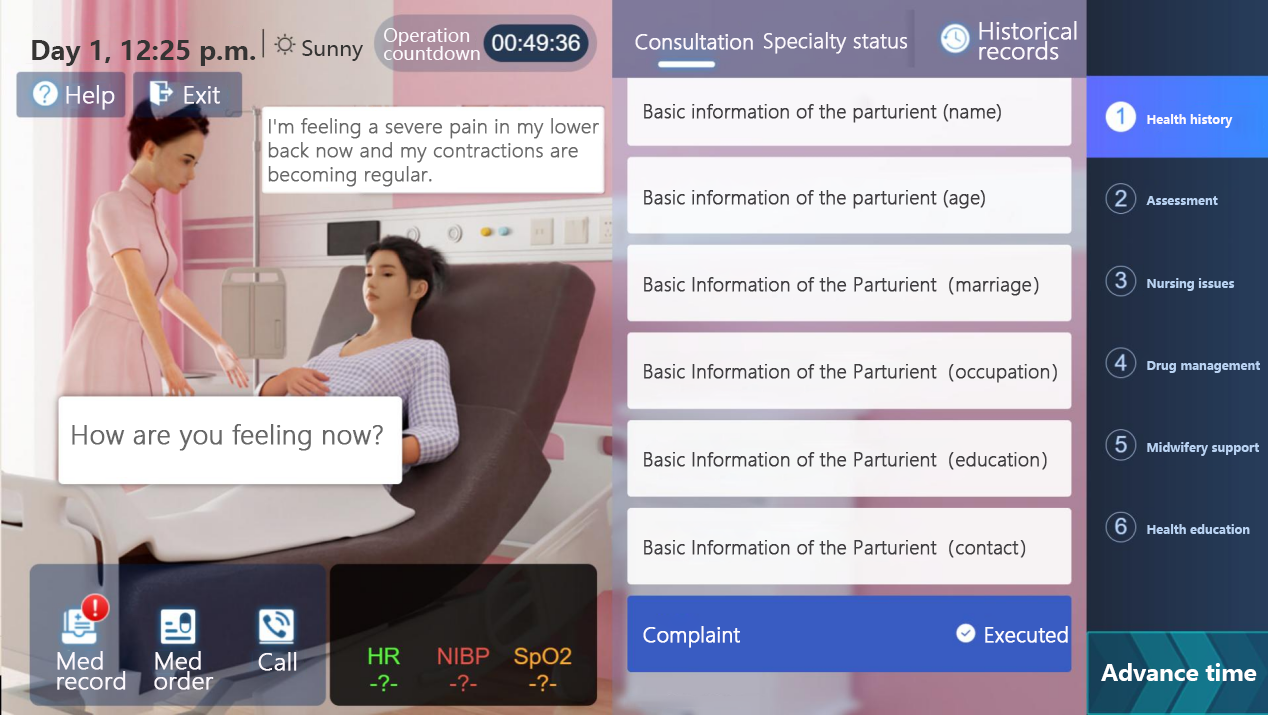


**Figure S2.** During the triage interaction, the virtual nurse conducts a comprehensive health history collection from the virtual laboring woman.


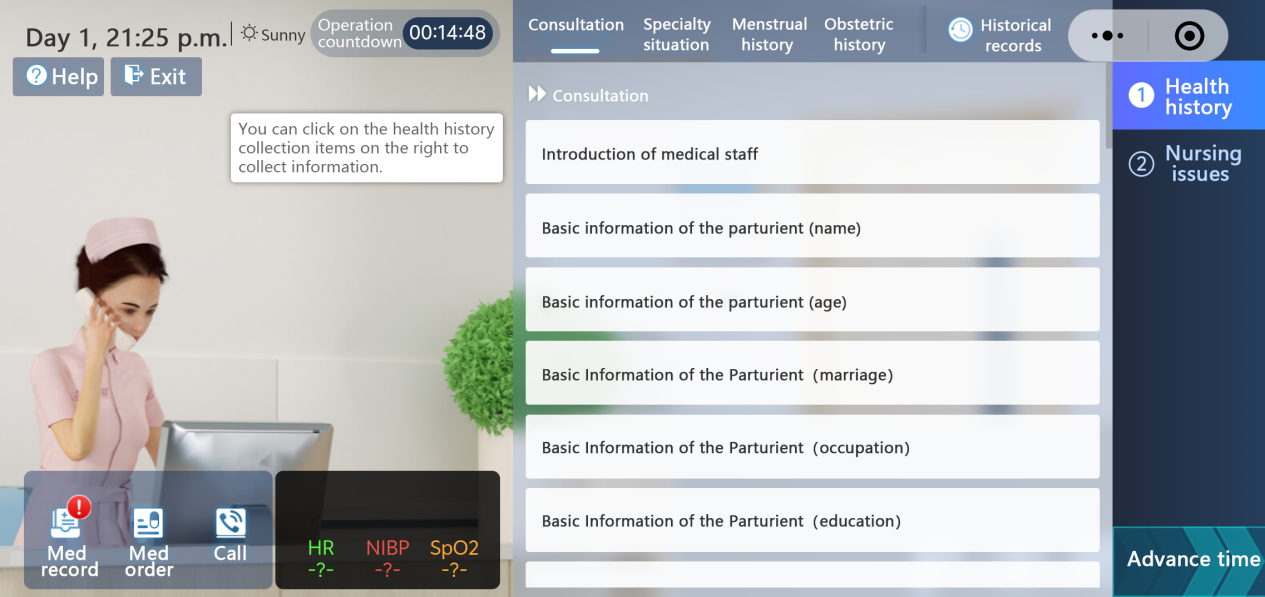


**Figure S3.** The virtual laboring woman’s specific clinical manifestation—integrating chief complaint and basic demographic information.


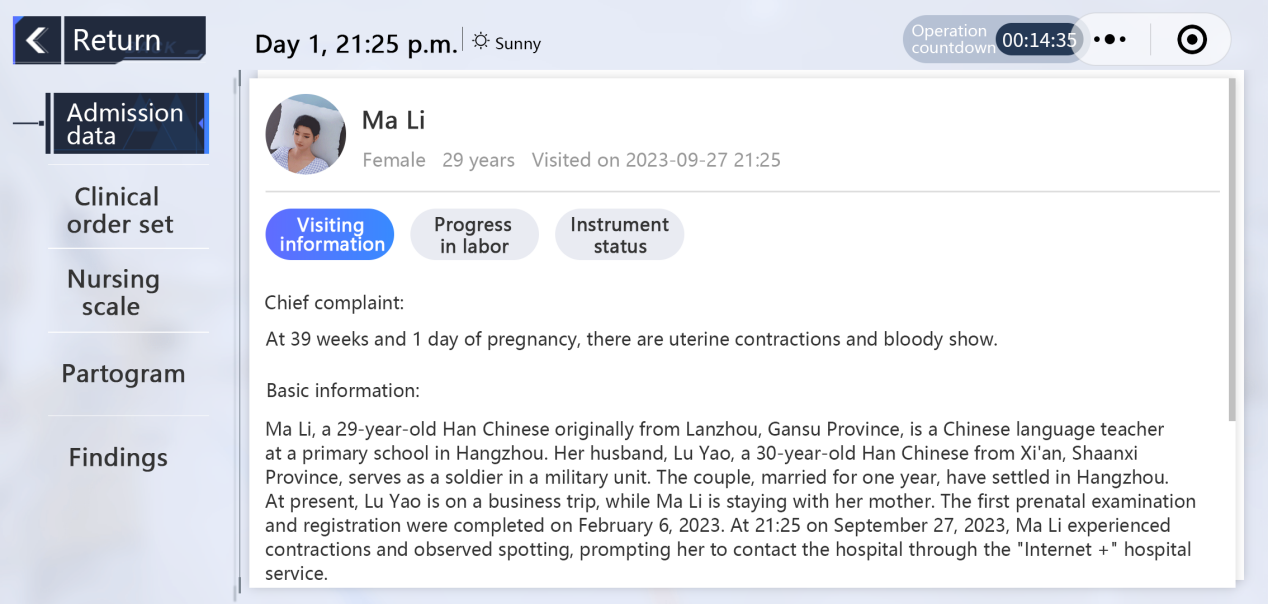


**Figure S4.** Upon completion of the training, the system automatically generates a comprehensive diagnostic score and an objective assessment of each student’s clinical competence.


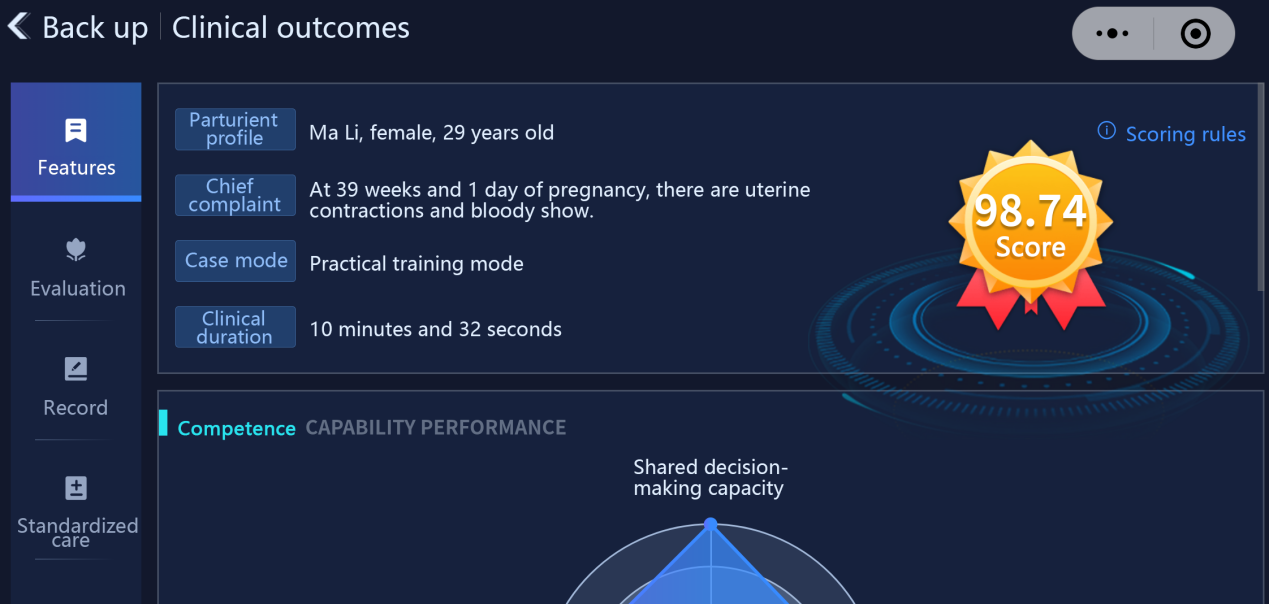

Supplement: Multimedia Appendix 2 [file jmir_v27i1e80951_app2.docx]
