# Supplementary material for: Use of a Nonimmersive Virtual Reality System for Clinical Thinking in Obstetric Nursing Education: Mixed Methods Study
Source: J Med Internet Res. 2025 Nov 24;27:e80951. doi: 10.2196/80951 (PMC12686860; doi:10.2196/80951)
Supplement: Multimedia Appendix 3 [file jmir_v27i1e80951_app3.docx]

**Content of quantitative data collection**

Dear Student：

Thank you for dedicating your valuable time to this study. The following outlines the quantitative data collection components for the study titled “Use of a Nonimmersive Virtual Reality System for Clinical Thinking in Obstetric Nursing Education: Mixed Methods Study”. These components include the General Information Questionnaire, the Clinical Thinking Ability Evaluation Scale (CTAES), and the Evaluation Instrument for Virtual Reality System (EIVRS). The purpose of this data collection is to gather basic demographic information and assess the effectiveness of the system in the context of online training. If you have any questions regarding the meaning or interpretation of any items, please do not hesitate to contact the researchers via the designated WeChat group. We promise to strictly keep all your data confidential and use it only for study. Your personal information will remain confidential and will not result in any adverse consequences. We sincerely appreciate your cooperation and support.

**General Information Questionnaire**

| Name： |  | School： |  |
| --- | --- | --- | --- |
| Gender： |  | Age： |  |
| Grade： |  | Case study experience： |  |
| Virtual reality learning experience： | | |  |

**Clinical Thinking Ability Evaluation Scale (CTAES)**

| Dimension | Item | **Option** |
| --- | --- | --- |
| Critical thinking | 1. Seeking the truth: When dealing with controversial and complex issues, one can analyze them from different perspectives to uncover the facts. | □Very poor □Poor □Average □Good  □Very good |
|  | 2. Open-mindedness: When facing problems, one can promptly understand others’ viewpoints, draw on excellent domestic and international experiences, and make objective evaluations of the issues. | □Very poor □Poor □Average □Good  □Very good |
|  | 3. Analytical ability: The diagnosis and nursing operations made are logical and systematic, and correct judgments can be made based on specific circumstances. | □Very poor □Poor □Average □Good  □Very good |
|  | 4. Self-confidence in critical thinking: having the ability to think precisely, being able to understand others’ thoughts in a timely manner, and possessing strong self-confidence. | □Very poor □Poor □Average □Good  □Very good |
|  | 5. The desire for knowledge: Acquiring new knowledge, exploring new things, and constantly accepting new challenges and difficult problems. | □Very poor □Poor □Average □Good  □Very good |
|  | 6. Cognitive maturity: The ability to recognize things (such as changes in a laboring woman’s condition) and the corresponding operations made. | □Very poor □Poor □Average □Good  □Very good |
| System thinking | 7. The ability to master basic and clinical knowledge and to identify clinical symptoms and signs. | □Very poor □Poor □Average □Good  □Very good |
|  | 8. Physical examination: The capacity to apply appropriate physical examination techniques to accurately detect and interpret various clinical signs, while ensuring effective laboring woman cooperation. | □Very poor □Poor □Average □Good  □Very good |
|  | 9. The utilization of auxiliary examinations: The ability to appropriately and effectively employ auxiliary diagnostic tools in clinical practice. | □Very poor □Poor □Average □Good  □Very good |
|  | 10. Medical history collection: A systematic and comprehensive process involving thorough laboring woman interviews and accurate documentation of clinical history. | □Very poor □Poor □Average □Good  □Very good |
|  | 11. Clinical condition monitoring: The systematic and timely identification of changes in a laboring woman’s clinical status. | □Very poor □Poor □Average □Good  □Very good |
|  | 12. During the collection of laboring woman data, various types of information can be systematically organized and classified within the clinician’s cognitive framework. | □Very poor □Poor □Average □Good  □Very good |
|  | 13. The capacity to systematically gather relevant information and analyze it from multiple perspectives in the context of diagnostic uncertainty regarding a laboring woman’s health condition. | □Very poor □Poor □Average □Good  □Very good |
|  | 14. As the labor process progresses, the individual is able to periodically synthesize the collected information and correlate it with their own thoughts. | □Very poor □Poor □Average □Good  □Very good |
|  | 15. When new clinical information emerges that suggests a potential health issue, it often necessitates a systematic review of previously collected data to evaluate its relevance and consistency with the identified condition. | □Very poor □Poor □Average □Good  □Very good |
|  | 16. Communication skills: The ability to fully gain the trust of laboring women, reasonable skills in communicating with laboring women, and the ability to obtain the required information. | □Very poor □Poor □Average □Good  □Very good |
|  | 17. Communication proficiency: The ability to systematically summarize medical history with clarity and logical organization, accurately describe clinical symptoms and physical findings, and employ appropriate terminology to facilitate precise laboring woman comprehension. | □Very poor □Poor □Average □Good  □Very good |
| Evidence-based thinking | 18. Research capability. | □Very poor □Poor □Average □Good  □Very good |
|  | 19. Awareness of evidence-based medicine. | □Very poor □Poor □Average □Good  □Very good |
|  | 20. The ability to translate clinical challenges and uncertainties encountered in medical practice into well-defined, specific, and researchable questions prior to implementing any interventions. | □Very poor □Poor □Average □Good  □Very good |
|  | 21. The ability to search for information and evidence using evidence-based medicine databases and the Internet. | □Very poor □Poor □Average □Good  □Very good |
|  | 22. The ability to critically evaluate the quality of retrieved literature and evidence in accordance with established evidence-based assessment criteria. | □Very poor □Poor □Average □Good  □Very good |
|  | 23. The ability to assess the validity of evidence (i.e., its alignment with objective facts). | □Very poor □Poor □Average □Good  □Very good |
|  | 24. The ability to synthesize the highest-quality research evidence (or information) with clinical expertise to facilitate its application in clinical practice. | □Very poor □Poor □Average □Good  □Very good |

The CTAES was developed by Song [1] to assess the clinical thinking ability of medical students. It has 3 dimensions—systematic thinking, critical thinking, and evidence-based thinking—and includes 24 items. Each item was assigned on a 5-point Likert scale ranging from 1 (very poor) to 5 (excellent). The raw total of 120 points was converted into a percentage for final reporting. Scores from 80 to 100 indicate excellent clinical thinking; scores from 60 to 79 indicate good ability; scores from 40 to 59 indicate average ability; scores from 20 to 39 suggest poor ability; and scores from 0 to 19 reflect very poor clinical thinking. The test-retest reliability score of this scale is 0.84, while the Cronbach *α* score is 0.91 [1].

**Evaluation Instrument for Virtual Reality System (EIVRS)**

| Dimension | Item | **Option** |
| --- | --- | --- |
| Interface design | 1. The interface employs a color scheme that is harmonious, clean, aesthetically pleasing, and appealing, thereby stimulating learning interest. | □Excellent□Good  □Average  □Poor |
|  | 2. The textual, pictorial, and other media elements are employed judiciously and coordinated effectively to highlight the key concepts and facilitate comprehension. | □Excellent□Good  □Average  □Poor |
|  | 3. The system exhibits a well-defined link hierarchy with clearly differentiated primary and secondary navigation levels. All hyperlinks function accurately, ensuring the absence of dead-end links. | □Excellent□Good  □Average  □Poor |
| Technical performance | 4. The navigation system is intuitively designed, user-friendly, and facilitates efficient access to the desired content. | □Excellent□Good  □Average  □Poor |
|  | 5. The system incorporates a diverse range of interaction methods and types, ensuring timely feedback to support collaborative learning, communication, and cooperative activities. | □Excellent□Good  □Average  □Poor |
| Learning content | 6. Learning content demonstrates the interdisciplinary integration and cross-pollination of knowledge across academic fields. | □Excellent□Good  □Average  □Poor |
|  | 7. The organizational structure of the system learning content is reasonable and accurate. | □Excellent□Good  □Average  □Poor |
|  | 8. The content of the system learning is relevant to the case content and richly diverse. | □Excellent□Good  □Average  □Poor |
|  | 9. The content of the system learning is suitable for the corresponding teaching objectives. | □Excellent□Good  □Average  □Poor |
|  | 10. The content of the systematic learning is suitable for the needs of learners. | □Excellent□Good  □Average  □Poor |
|  | 11. The system has a rich collection of case resources. | □Excellent□Good  □Average  □Poor |
|  | 12. The system’s case resources are diverse, appropriate, typical, and representative. | □Excellent□Good  □Average  □Poor |
| Learning function | 13. The system is reasonably modularized, structurally sound and fully functional. | □Excellent□Good  □Average  □Poor |
|  | 14. Systematic learning provides appropriate and effective assessment methods and features a wide variety of well-targeted questions. | □Excellent□Good  □Average  □Poor |
|  | 15. The system demonstrates significant effectiveness and outperforms traditional learning models. | □Excellent□Good  □Average  □Poor |

The EIVRS was developed by Chen [2] to assess the performance of virtual reality software in surgical nursing education. It has 15 items, and the overall score ranges from 0 to 10. It contains 4 domains—interface design (items 1-3; maximum score 1.5), technical performance (items 4-5; maximum score 1.5), learning content (items 6-12; maximum score 3), and learning function (items 13-15; maximum score 4). Each domain has weighted subitems. The following are the subitems along with their weights and coefficients: aesthetic style (5/1.25), media design (6/1.50), link design (4/1.00), navigation (6/1.50), interactivity (9/2.25), instructional material (18/4.50), resource quality (12/3.00), module division (8/2.00), learning assessment (12/3.00), and learning outcome (20/5.00). Subitem scores are calculated as (raw score × coefficient)/items per subitem; the composite score equals the actual total/10 (range 0-1), with >0.85 indicating excellent, 0.70-0.84 indicating good, 0.60-0.69 indicating moderate, and <0.60 indicating poor performance. The content validity index of the EIVRS is 0.80 [2].

**References**

[1] Song JY. A study on the evaluation index system of clinical thinking ability of medical students and demonstration. Qingdao University, 2015.

[2] Chen LL. The development and application of virtual clinical case software in surgical nursing. Taishan Medical College, 2013.

[3] Chen LL, Liu HX, Li L, et al. A randomized controlled trial of a virtual patient program for surgical nursing competency: development and evaluation. Chin J Nurs, 2014,49(02):226-229. [doi:10.3761/j.issn.0254-1769.2014.02.025]
