# Supplementary material for: Use of a Nonimmersive Virtual Reality System for Clinical Thinking in Obstetric Nursing Education: Mixed Methods Study
Source: J Med Internet Res. 2025 Nov 24;27:e80951. doi: 10.2196/80951 (PMC12686860; doi:10.2196/80951)
Supplement: Multimedia Appendix 4 [file jmir_v27i1e80951_app4.docx]

**Nonimmersive Virtual Reality System for Clinical Thinking in Obstetric Nursing (NIVRSCTON) in Obstetric Nursing in Online training**

***Reflective Journal***

| **Name:** |  |
| --- | --- |
| **School:** |  |
| **Grade:** |  |
| **Telephone:** |  |
| **Date of completion:** |  |

**Basic Requirements for Reflective Journal**

**1. Cover**

Please fill in all the information on the report cover completely.

**2. Detailed Requirements for Composing a Reflective Journal**

Please take the case in the Nonimmersive Virtual Reality System for Clinical Thinking in Obstetric Nursing (NIVRSCTON) as a whole and use the Bass Model as the framework to conduct a structured and comprehensive reflection on the entire process of this training. The detailed writing requirements are as follows.

| Reflection stage | | Reflection content |
| --- | --- | --- |
| 1 | Self-awareness | Identify a secure and tranquil space, direct your focus accordingly, and fully immerse yourself in the training environment generated by the NIVRSCTON. Attend to your emotional and cognitive responses, and document in writing what you perceive as most significant throughout the training process, accompanied by a concise rationale. |
| 2 | Description | Subsequently, please provide a detailed description of your experience without incorporating any analytical commentary. You may organize your account in chronological stages aligned with the case progression. You may conduct your analysis from the following perspectives. (1) What is your current location? Who is present with you? (2) What actions are you performing? What is the laboring woman doing? (3) How would you describe the training environment? What events are occurring within it? (4) What role do you assume in the training environment? What were the outcomes of the training? (5) What was the final outcome for the laboring woman? (6) Which critical issues require particular attention throughout the entire training process? |
| 3 | Reflection | Engage in a reflective exploration of your thoughts and emotions, and conduct a comprehensive consideration of your training experience, including your present cognitive and affective responses. You may structure your reflection in chronological stages aligned with the case progression. You may conduct your analysis from the following perspectives. (1) What were your primary objectives in this training?  (2) What motivated your decisions and actions throughout the training? (3) What potential consequences might your actions have on yourself, the laboring woman, and her family? (4) How might the laboring woman perceive or interpret your actions? (5) What were your internal thoughts and considerations during the training? (6) What emotional responses did you experience within that contextual environment? (7) How did the eventual outcome of the event impact your emotional state? |
| 4 | Impact or cognition | Examine this experience from multiple perspectives to identify the internal (personal) and external (environmental or interpersonal) factors that shaped your decisions and actions. You may conduct your analysis from the following perspectives. (1) What internal factors influenced your decisions and actions? (2) What external factors influenced your decisions and actions? (3) What theoretical or practical knowledge influenced or should have influenced your decision-making and behavioral responses? |
|  |  | You may conduct your analysis from the following internal and external perspectives.  (1) Empirical: science.  (2) Moral: moral cognition.  (3) Personal: self-awareness, past experience, intuitive.  (4) Aesthetic: creativity, practical art, intuition.  (5) Socio-political: dominant ideology, leading theories influencing healthcare.  (6) Unknown: uncertainty.  (7) Technocracy (expert politics), humanism, holistic approach.  (8) Cognitive ways of construction, connection and separation. |
| 5 | Evaluation (analysis) | The first 4 stages have examined the experience from multiple perspectives and provided comprehensive information to support your critical reflection. In this stage, you are expected to evaluate: which aspects of this training were effective? Which encountered challenges? Could alternative strategies have been implemented? What specific actions could have been taken to improve outcomes? You may conduct your analysis from the following perspectives. (1) What aspects of the training proceeded effectively, and what skills or actions contributed to these positive outcomes? (2) What challenges were encountered, and what factors contributed to the gaps between expected and actual outcomes? (3) How did the interventions or decisions you made impact the outcomes for the laboring woman and newborn? (4) What strategies could enhance your performance and decision-making in similar future scenarios? (5) Are there alternative interventions that could have been applied? How might these have influenced the outcomes for the laboring woman and newborn? |
| 6 | Learning (integration) | You may describe your learning reflections from the following perspectives. (1) Describe your current emotional state and reflective feelings regarding the training experience. (2) What key insights and professional competencies have you gained through this training? (3) How does this training align with or expand upon your previous learning experiences in clinical nursing? (4) What specific strategies or interventions did you implement to support both the laboring woman and your own professional development? (5) On a personal level, has this training enhanced your understanding of clinical practice? Provide detailed reflections. (6) What critical insights or inspirations have emerged regarding obstetric nursing practice as a result of this training? (7) In what ways has this training influenced or transformed your personal beliefs and professional perspective on obstetric clinical nursing? (8) How might this training influence your future academic and clinical training? (9) In what ways has this training contributed to your professional growth and readiness to assume the role of an obstetric nurse? |

Please adhere to the following formatting guidelines when completing the reflective journal: level 1 headings should be formatted in size 4 Song font, 1.5 line spacing, and bold; level 2 headings and subheadings should use size 5 Song font, 1.5 line spacing, and bold; the main text should be in size 5 Song font, 1.5 line spacing, with a first-line indent of two characters.
