# Supplementary material for: Use of a Nonimmersive Virtual Reality System for Clinical Thinking in Obstetric Nursing Education: Mixed Methods Study
Source: J Med Internet Res. 2025 Nov 24;27:e80951. doi: 10.2196/80951 (PMC12686860; doi:10.2196/80951)
Supplement: Multimedia Appendix 5 [file jmir_v27i1e80951_app5.docx]

Themes extracted from students’ reflective journals following the Nonimmersive Virtual Reality System for Clinical Thinking in Obstetric Nursing (NIVRSCTON) training.

| Themes and subthemes | Quotes |
| --- | --- |
| 1. Reflection on practical actions |  |
| 1.1 Maintaining calm | - *“When facing new challenges and emergencies, I might feel a bit nervous and excited, but at the same time, I will remain calm and focus on making the right decisions.”* [S10] |
| 1.2 Assessment and support throughout the labor | - *“Health assessments should be integrated throughout the entire labor process, as this can assist with the development of appropriate support plans.”* [S7] |
| 1.3 Humanistic care | - *“Humanistic care is essential to effectively alleviate negative emotions in laboring woman by providing professional psychological counseling and emotional support.”* [S5] |
| 1.4 Establishing partnerships | - *“A trusting, cooperative relationship empowers laboring women and their families to navigate childbirth challenges more effectively.”* [S6] |
| 1.5 Avoiding medical risks | - *“If a laboring woman has retained placenta that goes undetected, it can certainly result in medical malpractice.”* [S32] |
| 2. Reflection on practice ability |  |
| 2.1 Promoting the integration of theoretical knowledge with practical application | - *“In past studies, we mostly passively or actively accepted the theoretical knowledge taught by teachers, but our understanding was often not deep enough, mostly just general summaries. In this practical training, I will organize the knowledge of each key link to form a clear overall idea to better guide operations.”* [S14] |
| 2.2 Enhancing adaptability in training | - *“This time, it facing the identification and management of an entire production process, and the time is quite tight, requiring my clinical response speed and accuracy.”* [S9] |
| 2.3 Strengthening evidence-based thinking | - *“I realized that we should not rigidly adhere to standard textbook procedures. Instead, we need to analyze each labor woman’s condition critically and make correct immediate decisions based on the specific case.”* [S42] |
| 2.4 Fostering professional identity | - *“After the training, I am more confident about becoming an obstetric nurse in the future.”* [S9] |
| 2.5 Facilitating team collaboration | - *“During the training, the team collaborated closely to accomplish all assigned tasks and achieved highly commendable outcomes. This experience further reinforced my appreciation for teamwork and the importance of a collaborative spirit.”* [S10] |
| 3. Reflect on the multidimensional factors influencing practice overview |  |
| 3.1 Internal factors | - *“Personal values, moral principles, and emotional states can significantly influence decision-making processes and subsequent actions.”* [S10] - *“My mastery of theoretical knowledge affects my practical training.”* [S2] |
| 3.2 External factors | - *“As the system was unable to provide immediate feedback regarding the correctness of the practice, nor could it inform me about the conditions of the laboring woman and the newborn immediately following the procedure, I experienced significant anxiety and tension throughout the practice.”* [S23] - *“Because I am not yet proficient with the system, my performance has been suboptimal. Therefore, I request multiple training sessions on system operation before the formal training to reduce any anxiety arising from unfamiliarity.”* [S30] |
