# Supplementary material for: Use of a Nonimmersive Virtual Reality System for Clinical Thinking in Obstetric Nursing Education: Mixed Methods Study
Source: J Med Internet Res. 2025 Nov 24;27:e80951. doi: 10.2196/80951 (PMC12686860; doi:10.2196/80951)
Supplement: Multimedia Appendix 6 [file jmir_v27i1e80951_app6.docx]

Integration of quantitative and qualitative results.

| Main quantitative findings | Main qualitative findings | | | Integrated findings |
| --- | --- | --- | --- | --- |
|  | Themes | Subthemes | Representative quotes |  |
| System thinking  44.51 (6.24)^a^ | Reflection on practical actions | Assessment and support throughout the labor | S1: “We have performed a series of comprehensive nursing assessments, diagnoses, health education, and support for the laboring woman. These measures are aimed at ensuring the safety of both the laboring woman and newborn.” | - Confirmation:   The training effectively enhanced students’ system thinking, improved their practical competencies in assessment and guidance, and promoted the integration of theoretical knowledge with practical application. |
|  | Reflection on practice ability | Promoting the integration of theoretical knowledge with practical application | S32: “In the dynamic and continuous process support, I need to promptly identify problems and provide assistance. Applying the knowledge I have learned flexibly to the practical training helps to make up for the shortcomings of traditional training and enhances my abilities.” |  |
| Critical thinking  24.77 (3.11)^a^ | Reflection on practice ability | Enhancing adaptability in training | S2: “The system covers the abnormal delivery process. I need to identify and handle the problems within a limited time, which helps to exercise my adaptability and clinical thinking.” | - Confirmation:   The training enhanced students’ critical thinking and strengthened their ability to adapt to changing situations. |
| Evidence-based thinking  27.31 (4.61)^a^ | Reflection on practice ability | Strengthening evidence-based thinking thinking | S6: “In the classroom, we learned that after the fetus is delivered, assistance is needed for the repositioning of the fetal head and external rotation. However, after this training session, I learned from the latest guidelines that there are certain points regarding not rushing to assist with the repositioning of the fetus, external rotation, and delivery of the shoulders.” | - Confirmation:   Students demonstrated significant improvement in evidence-based thinking after the training. |
|  | Cultivate professional qualities | |  |  |
|  | Reflection on practical actions | Maintaining calm | S8: “Paying attention to one’s own emotions and state to ensure remaining calm and focused is also key to giving confidence to the laboring woman and her family.” | - Expansion:   The training effectively enhanced students’ practical behaviors in maintaining calm, providing humanistic care, establishing partnerships, and avoiding medical risks. |
|  |  | Humanistic care | S9: “When experiencing pain, the laboring woman receives my empathetic support, along with evidence-based labor analgesia strategies to effectively relieve her discomfort and enhance her confidence in the birthing process.” |  |
|  |  | Establishing partnerships | S8: “Foster a strong partnership with the woman and her family to ensure that the woman receives comprehensive understanding and support throughout the delivery process.” |  |
|  |  | Avoiding medical risks | S41: “Omitting any step in a virtual reality scenario may not result in significant consequences. However, it can help me avoid medical risks in real obstetric environments.... I know in real-world clinical practice, such seemingly minor errors can have devastating, irreversible consequences for both the laboring woman and newborn.” |  |
|  | Reflection on practice ability | Facilitating team collaboration | S5: “I need to maintain close communication and collaboration with members of the medical team... Ensure the safety of both the laboring woman and the newborn.” | - Expansion:   Students reported that the training enhanced team collaboration and strengthened professional identity. |
|  |  | Fostering professional identity | S6: “The training enabled me to grasp critical concepts, such as the recommendation in the latest clinical guidelines to avoid hastening shoulder delivery. This experiential learning has fundamentally reshaped my clinical understanding and is expected to yield lasting positive effects on my future academic pursuits and professional growth.” |  |
|  | Reflection on the multidimensional factors influencing practice | Internal factors (knowledge, skills and a sense of responsibility) | S2: “Scientific and authoritative guidelines, textbooks, and skills are the primary factors influencing my clinical decision-making and actions.”  S4: “Pressure and a sense of responsibility will compel me to maintain a calm demeanor, systematically analyze the circumstances surrounding the laboring woman, and deliver appropriate midwifery support.” | - Expansion:   The internal factors influencing students’ performance and decision-making during training included their knowledge, skills, and sense of responsibility, among others. |
| Learning function  0.85 (0.15)^a^ |  |  |  | - Expansion:   Students rated the learning function and content highly. |
| Learning content  0.83 (0.14)^a^ |  |  |  |  |
| Interface design  0.82 (0.15)^a^ | Reflection on the multidimensional factors influencing practice | External factors (unfamiliarity with system operation; the need for optimization of the system’s performance) | S7: “The system interface encompasses a variety of functions and content. Occasionally, I inadvertently select information that is not pertinent to the current stage of assessment... Furthermore... It would be advantageous to incorporate both prompt subtitles and audio features.” | - Confirmation:   Students gave relatively low ratings to the system’s interface design due to cluttered interface elements, lack of auditory cues, and the need for performance optimization. |
| Technical performance  0.82 (0.15)^a^ | Reflection on the multidimensional factors influencing practice | External factors (the degree of authenticity of the virtual reality training environment; the sense of urgency created by the countdown) | S29: “The operational workflow of the virtual reality system differs substantially from that of laboratory training. When encountering a highly simulated and clinically relevant training environment, I feel rather apprehensive.”  S20: “The countdown within the system induced a significant level of tension in me during the training.” | - Confirmation:   Students gave relatively low ratings to the system’s technical performance, primarily due to the degree of authenticity of the virtual reality training environment, as well as the sense of urgency created by the countdown feature. |

^a^ representative mean (SD)
